# Evaluation of Malolactic Bacteria Associated with Wines from Albariño Variety as Potential Starters: Screening for Quality and Safety

**DOI:** 10.3390/foods9010099

**Published:** 2020-01-17

**Authors:** Jacobo López-Seijas, Belén García-Fraga, Abigail F. da Silva, Xavier Zas-García, Lucía C. Lois, Ana Gago-Martínez, José Manuel Leão-Martins, Carmen Sieiro

**Affiliations:** 1 Department of Functional Biology and Health Sciences, Microbiology Area, University of Vigo, Lagoas–Marcosende, 36310 Vigo, Spain; lopezseijasjacobo@gmail.com (J.L.-S.); belengarciafraga@gmail.com (B.G.-F.); abi_fernandez@hotmail.com (A.F.d.S.); 2 Department of Research & Development of Cellar “Condes de Albarei”, Lugar A Bouza 1, 36639 Cambados, Spain; gerencia@condesdealbarei.com (X.Z.-G.); lclois@yahoo.es (L.C.L.); 3 Department of Analytical and Food Chemistry, University of Vigo, Lagoas–Marcosende, 36310 Vigo, Spain; anagago@uvigo.es (A.G.-M.); leao@uvigo.es (J.M.L.-M.)

**Keywords:** lactic acid bacteria, wine, malolactic fermentation, selected strains

## Abstract

The biodiversity of lactic acid bacteria in musts and wines of Albariño variety has been studied. The identification of species was addressed through a combination of biochemical and genetic methods (API^®^ 50 CHL test, 16S rDNA and *recA* gene sequences, Amplified Ribosomal DNA Restriction Analysis -ARDRA- and 16S-26S intergenic region analysis). The results grouped the isolates into six species predominating those of the genus *Lactobacillus* and showing a typical biogeographical distribution. Among sixteen strains evaluated, eight of them showed malolactic activity. The study of the presence of genes *hdc*, *odc,* and *tdc*, along with the LC/MS-MS analysis of biogenic amines in wine, showed five strains lacking aminogenic ability. The absence of the *pad* gene in the above-mentioned strains discards its ability to produce volatile phenols that may adversely affect the aroma. Finally, all malolactic strains showed β-glucosidase activity so that they could contribute to enhance and differentiate the aromatic profile of Albariño wines.

## 1. Introduction

The complex process of wine-making first consists of a fermentation carried out by yeasts that mainly convert sugars into ethanol. In some wines this fermentation is followed by a second transformation known as malolactic fermentation (MLF) conducted by lactic acid bacteria (LAB). The malolactic fermentation is a biological process of deacidification of wines comprising the decarboxylation of malic acid into lactic acid and CO_2_ leading to a reduction in the total acidity of the wine and a slight increase in pH. If this second fermentation is carried out by appropriate strains, it may also help to increase the microbiological stability of the product and to enhance its organoleptic characteristics, in particular its aromatic profile [1].

The malolactic fermentation takes place mostly in red wines, and very exceptionally in some acidic whites. Different previous studies have revealed the presence of different species and strains of LAB in spontaneous malolactic fermentations although in most cases the predominant species is *Oenococcus oeni* [2,3,4,5].

Although the MLF can occur spontaneously, this does not always happen and, in many cases, it may be difficult to achieve [1]. Moreover, due to the diversity of LAB strains which may be present, the most appropriate fermentation is not always carried out. This may involve different risks for the quality of the product such as a significant increase in the volatile acidity or the formation of undesirable metabolites [1,6]. To help avoid these problems and to carry out a controlled process, starters of LAB are marketed. These consist of selected strains, mostly of *O. oeni* species, traditionally considered the best adapted to the hostile environment of wine [7]. However, these malolactic starters often cannot be implanted in the different types of wine where they can be used, and do not allow a successful development of the process [8]. That is why manufacturers are making great efforts to provide the sector with different types of starters that are more robust, and based on different selected strains of *O. oeni*, the combination of several strains of this species in the same starter (up to four) or, more recently, combinations of *O. oeni* and other species, in particular *Lactobacillus plantarum*. However, many producers are still reporting problems in the implantation of the starters and in the achievement of a successful malolactic fermentation [9]. Studies have shown that the success of malolactic starters depends on the strain used, which in turn is influenced by several factors including the adaptation to wine-specific conditions in each wine-growing area [3]. That is why numerous studies support the idea of exploring the biodiversity of lactic acid bacteria associated with each wine-growing area to select strains better adapted to each type of wine and with the best features to carry out MLF [5,7,10]. Biogeography studies, which show that microbial communities associated with a wine region depend on the environment and the vine variety, corroborate this idea [11]. Moreover, although uncommon, some research has shown the prevalence of malolactic species different from *O. oeni* in certain wine regions, as well as a growing interest in these to be used as starters given their favorable characteristics [12,13,14]. A recent starter formulation made by Lallemand Inc., based on a selected *L. plantarum* strain, confirms this evidence.

The wine region of Val do Salnés, belonging to the Appellation of Origin Rías Baixas in Spain, has an Atlantic climate, warm and humid where mostly Albariño, a white grape variety of *Vitis vinifera*, is grown. About 25,000,000 kg of this type of grape are harvested each year in the above-mentioned region. On many occasions and due to excess of acidity, the wines obtained with this variety in this area, unlike those made from the same variety in other areas, must complete the MLF. However, frequently, the successful development of spontaneous MLF or the implantation of commercial starters in these wines has been difficult to achieve. These reasons have justified the approach of this study on the biodiversity of LAB associated with wines of this region, as well as their characterization in order to determine their potential for the development of new indigenous starters.

## 2. Materials and Methods

### 2.1. Sampling and Lactic Acid Bacteria (LAB) Isolation

Samples of Albariño musts and wines were taken during two consecutive vintages (2007 and 2008) in the Val do Salnés region in Spain (see supplementary Figure S1). The winery Condes de Albarei, which is considered representative of the area, was selected because it works as a cooperative society and receives grapes from different sub-areas of the region. Grapes from the sub-areas are mixed to obtain the must used to fill the fermentation tanks. Sampling was randomly done in the cellar. One liter samples were taken aseptically from eight tanks at different stages: must (with 25 mg/L of free SO_2_), alcoholic fermentation and malolactic fermentation (carried out at a controlled temperature of 19–21 °C), transported to the laboratory at 4 °C, and processed for bacteria isolation.

Each sample was concentrated by centrifugation (22,000× *g*) and resuspended in 10 mL of sterile saline solution or diluted in the same solution and plated onto media MLO (Scharlab Chemie SA, Barcelona, Spain) and MRS (de Man, Rogosa and Sharpe) (Panreac AppliChem, Barcelona, Spain) supplemented with 10% (*v*/*v*) tomato juice. Cycloheximide (100 mg/L) was added for inhibiting yeast growth. Plates were incubated under strict anaerobe conditions (Anaerocult^®^ A, Merck, Barcelona, Spain) for 7 days at 30 °C. Colonies were sub-cultured onto the same medium for reisolation and isolates were stored at −80 °C with glycerol (30%).

### 2.2. Reference Strains

The reference strains, from the Spanish Type Culture Collection (CECT), used in this study were *Lactobacillus hilgardii* CECT 4786; *Oenococcus oeni* CECT 217; *Pediococcus damnosus* CECT 793; *Lactobacillus casei* CECT 475; *Lactobacillus rhamnosus* CECT 288; *Lactobacillus paracasei* subsp. *tolerans* CECT 4175; *Lactobacillus pentosus* CECT 4023; *Lactobacillus plantarum* CECT 220; *Lactobacillus paraplantarum* CECT 5787; *Lactococcus lactis* CECT 185; *Lactobacillus helveticus* CECT 403; *Lactobacillus rhamnosus* CECT 288.

### 2.3. Identification of LAB Isolates

Species identification and differentiation was accomplished by a combination of biochemical and molecular methods. Gram staining, catalase test, microscope morphology, and assimilation of carbon sources by the API^®^ 50 CHL (bioMérieux^®^ SA, Madrid, Spain) were determined for the biochemical identification of the isolates. Bacterial DNA isolation for molecular identification of the isolates was carried out using the Wizard^®^ Genomic DNA Purification Kit (Promega, Madison, WI, USA). The 16S rDNA region was PCR-amplified according to Weisburg et al. [15]. After gel purification (FavorPrep^™^ GEL/PCR Purification Kit, Favorgen Biotech Corp., Vienna, Austria), DNA fragments were sequenced and aligned with those in GeneBank using the Basic Local Alignment Search Tool (BLAST) 2.0 [16] in order to determine the closest known sequences. For restriction analysis of the amplified 16S rDNA region (16S-ARDRA), the PCR product was digested with the following restriction enzymes: *Mse*I, *Bfa*I, *Alu*I, *Hae*III, and *Btg*ZI (New England Biolabs^®^ INC., Frankfurt am Main, Germany), according to Rodas et al. [2]. Fragments were resolved by 1.5% agarose gel electrophoresis. The intergenic spacer region (ISR) between the 16S and 23S genes was also amplified by PCR following the method previously described by Moreira et al. [17]. Product purification and analysis of the restriction fragments obtained with *Vsp*I enzyme (Fermentas, Ontario, Canada) [17] were done as indicated above. *RecA* amplification, by a multiplex PCR, was performed as previously described by Torriani et al. [18]. *RecA* gene was also amplified for sequencing as described by Torriani et al. [18]. Analysis of the amplified DNA was performed as previously described.

### 2.4. Characterization of LAB Strains

#### 2.4.1. Microvinifications and Wine Analysis

In order to evaluate the malolactic capacity of the isolated strains, controlled malolactic fermentations were carried out at laboratory scale in glass flasks containing 100 mL of wine from the Albariño variety also collected from the cellar studied (ethanol content of 12% (*v*/*v*), residual sugars <2 g/L, pH 3.8, total acidity 6.28 g/L, malic acid 4.8 g/L, and total SO_2_ 75 mg/L). Wine was previously centrifuged at 9800× *g* for 15 min and filtered through 0.22 μm filters. Microfermentations were inoculated using an exponential culture of each assayed LAB at a final cell density of 10^6^ cells/mL and incubated at 20 °C for 15 days. Wines were then filtered and stored for further analysis. Uninoculated fermentations were included as control. The assays were done in duplicate.

All chemical parameters of wine, including malic, lactic, and acetic acid, were analyzed using a FOSS FTIR Winescan.

#### 2.4.2. Biogenic Amines (BAs)-Forming Capacity

##### Molecular Determination of BAs Formation Capacity

The presence of the genes *hdc*, *tdc*, and *odc*, coding for histidine, tyrosine, and ornithine decarboxylase enzymes, respectively, was assessed following the method described by De Las Rivas et al. [19]. Although the authors performed this protocol as a multiplex PCR, we carried it out independently for each gene.

##### Quantitative Determination of BAs

1. Chemicals and standards

Water and acetonitrile (LC/MS Grade) were purchased from Panreac (Barcelona, Spain). Methanol (HPLC grade) and hydrochloric acid (37% purity) analytical grade were purchased from Merck (Barcelona, Spain). Formic acid (98–100% purity), ammonium formate (≥99% purity), tyramine (TYR, HOC_6_H_4_CH_2_CH_2_NH_2_, MW = 137.18 g/mol, 99% purity), putrescine (PUT, NH_2_(CH2)_4_NH_2_, MW = 88.15 g/mol, 99% purity), and histamine dihydrochloride (HIS, C_5_H_9_N_3_ 2HCl, MW = 184.07 g/mol, ≥99% purity) were purchased from Sigma-Aldrich (Madrid, Spain).

2. Preparation of standard solutions and wine samples

Stock standard solutions with concentrations of 10 mg/mL of each biogenic amine (TYR, PUT, and HIS) were prepared in 1% *v/v* of HCl and stored at 4 °C. Working standard solutions (contains TYR, PUT, and HIS) were prepared weekly in aqueous methanol (50% *v*/*v*) in concentrations ranging from 0.25 to 10 µg/mL.

Wine samples were diluted in mobile phase and filtered through a PVDF filter with a pore size of 0.2 µm (PVDF Membrane, Millipore, Cork, Ireland) prior to analysis.

3. UHPLC-MS/MS analysis

Chromatographic separation was performed according to Self et al. [20] using an Agilent 1290 Infinity UHPLC system. Biogenic amines were separated using hydrophilic interaction chromatography (HILIC) column (Aquity UPLC^®^ BEH Amide column, Lot number 0104191311, 2.1 mm × 50 mm, 1.7 µm) from Waters at 40 °C. Mobile phase A was 100% water containing 30 mM of ammonium formiate and 1 mL of formic acid and B was 100% acetonitrile. MS detection was performed using an Agilent G6460A triple quadrupole mass spectrometer equipped with an Agilent Jet Stream ESI source. Source and interface conditions were optimized for the analysis of biogenic amines in positive ionization mode and adjusted to gain the best sensitivity for the three biogenic amines previously mentioned.

4. Quantitation parameters

Calibration was carried out using standard solutions of a mixture of putrescine, tyramine, and histamine biogenic amines in a range from 0.25 to 10 µg/mL. A blank control was injected before and after the calibration sets in order to check the potential column overload.

Signal-to-noise ratio (S/N = 3 and S/N = 10) was used for the calculation of the limit of detection (LOD) and limit of quantitation (LOQ), respectively, using the lowest calibrant solution prepared in solvent and based on triplicate injection (Table S1).

#### 2.4.3. β-Glucosidase Activity

β-glucosidase activity of LAB isolates was determined on culture biomass following the protocol described by Grimaldi et al. [21], using *p*-nitrophenyl-β-D-glucopyranoside (*p*-NPG) as substrate. Activity was estimated at 18 °C and pH 4 (achieved with 100 mM McIlvane buffer). One unit of activity was defined as mmols of *p*-nitrophenol liberated per minute and per mg of cell dry weight.

All the analytical determinations were done in triplicate.

#### 2.4.4. Phenolic Acid Decarboxylase Production Capacity

The presence of *pad* gene (encoding a phenolic acid decarboxylase), which was correlated with volatile phenol production, was established using the method described by Mtshali et al. [22]. The *pad* gene was amplified by PCR using the degenerate primers PAD-1 (5′-AARAAYGAYCAYACYRTTGATTACC-3′) and PAD-3 (5′-TTCTTCWACCCAYTTHGGGAAGAA-3′). Fragments of the expected size were resolved on a 2% agarose gel.

## 3. Results and Discussion

### 3.1. Isolation and Identification of the Isolates: Ecological Distribution

For two consecutive vintages the isolation of malolactic bacteria was carried out in fermentations performed with *Vitis vinifera* Albariño, a variety grown in the region of Val do Salnés, Spain. Samples were taken from grape must (M), alcoholic fermentation (AF), and malolactic fermentation (MLF). Among all isolates as potential lactic acid bacteria, the Gram-positive/catalase-negative were selected. In total, 35 isolates were obtained (UVI-1–UVI-35). This frequency of isolation can be classified as low or very low when compared with data from other wine-making areas studied by Ruiz et al. [3], although low frequency isolation was also reported in other producing regions by Valdés La Hens et al. [14].

The ability to assimilate 49 carbohydrates, by 35 isolates, was determined by API^®^ 50 CHL test, compared with different reference strains of malolactic species. The results did not permit conclusive identification of all isolates (Table 1), but since it was described in a previous study by Testa et al. [13], a first grouping was allowed. Also, API^®^ 50 CHL test revealed differences in carbohydrate assimilation among most of the isolates (supplementary Figure S2). For this reason the molecular identification continued with all of them. Different molecular methods have been successfully used for identification of lactic acid bacteria present in wine. These include the 16S rDNA [23], and *rec*A [18,24] gene sequences, the 16S-Amplified Ribosomal DNA Restriction Analysis (ARDRA) [2,23], and the 16S–23S intergenic region (ISR) analysis [17]. The application of these techniques to the isolates, along with the patterns obtained with the reference strains and the comparison with the sequences deposited in databases, allowed their definitive identification as shown in Table 1 (detailed identification data are shown in supplementary Figures S3 and S4).

**Figure 1:**
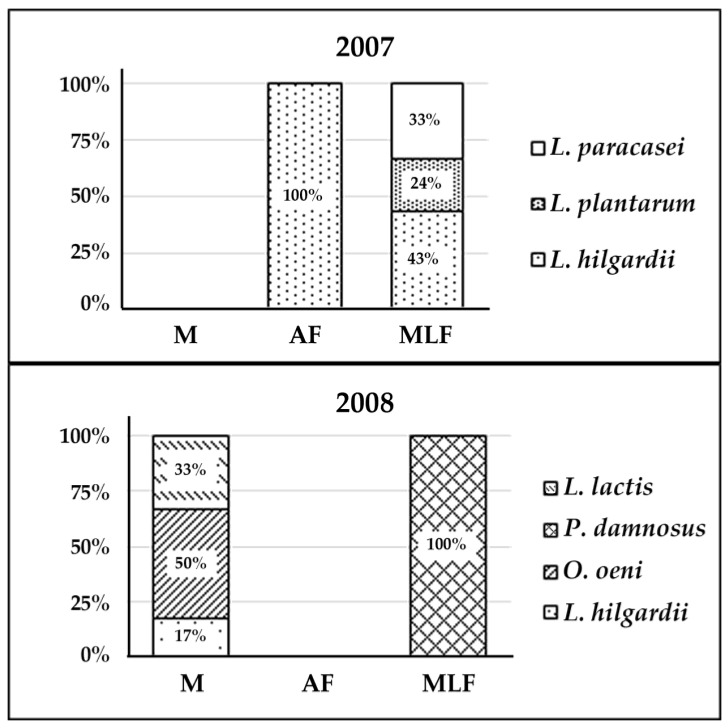
Annual distribution and frequency (%) of species of isolated lactic acid bacteria. M: must; AF: alcoholic fermentation; MLF: malolactic fermentation.

The analysis of the data shows a pattern of isolates different for each of the two years studied (Figure 1) which was also previously described in other wine regions [3]. Considering the total isolates obtained as a whole during the two years, 34.3% (12) belonged to species *Lactobacillus hilgardii*, 20% (7) to *Lactobacillus paracasei*, 17.2% (6) to *Pediococcus damnosus*, 14.3% (5) to *Lactobacillus plantarum*, 8.6% (3) to *Oenococcus oeni,* and 5.7% (2) to *Lactococcus lactis*. A total of six different species, which could be estimated as considerable diversity given the low number of isolates obtained, were identified. 

The results show an endemic microbiota for the Albariño variety grown in the region of Val do Salnés. Unlike what has been described for most wine regions traditionally studied [3,4,25], LAB predominant species in wines of the Albariño variety grown in this region belong to the genus *Lactobacillus* and not to species *O. oeni*, which in any of the years analyzed has not yet been found in MLF. Our data coincide with those of some wine regions recently studied for which the species of the genus *Lactobacillus* were also described as predominant [13,23,26]. The data are consistent with those shown by biogeography studies that show that microbial communities associated with a wine region depend on the environmental conditions, annuity, and vine variety [11].

### 3.2. Oenological Characterization

The relatively low frequency of LAB isolation in the wine region studied could be related to the observed difficulties in the achievement of MLF in wines of this area. Furthermore, the fact that neither the *O. oeni* species is predominant in this region, nor that it has been isolated in malolactic fermentation, questions the adequacy of existing commercial starters to conduct malolactic fermentation in Albariño wines of this region. Moreover, as shown, the success of a starter depends on the strain used and its adaptation to the specific conditions of each wine [3]. On the other hand, as occurs with yeasts [27] the use of indigenous LAB starters is of growing interest due to its proven ability to enhance the wine’s regional identity [9]. For these reasons, in the present work, the potential of LAB isolated in this region to be used in inducing MLF was evaluated. To this end, in the first selection, the isolates which showed better growth in laboratory media and wine, and that which presented a different profile of assimilation of sugars in the API^®^ 50 CHL test, were chosen: one strain of *L. plantarum* species (UVI-9), two strains of *O. oeni* (UVI-25), three strains of *L. paracasei* species (UVI-2, UVI-3, and UVI-5), and ten strains of *L. hilgardii* species (UVI-16, UVI-13, UVI-26, UVI-18, UVI-14, UVI-21, UVI-23, UVI-20, UVI-17, and UVI-15).

All isolates belonging to the species *P. damnosus* were discarded because its weak growth failed to produce suitable cell densities for inoculation of microvinifications. With the selected strains, a trial of microvinification in Albariño wine of the region studied was carried out and the results on the consumption of malic acid and production of lactic and acetic acid are shown in Table 2. One can observe a significant variability among the strains tested in terms of their ability to consume malic acid (0–78%), confirming that it is a property of the strain and not of the species. Two strains of the *L. paracasei* species stand out for their malolactic ability (UVI-2 and UVI-5) and six strains of the *L. hilgardii* species (UVI-14, UVI-17, UVI-18, UVI-20, UVI-21, and UVI-23). It is also interesting to note that strains that showed malolactic activity did not significantly increase the volatile acidity of the wines.

Malolactic species isolated in Albariño variety grown in the Val do Salnés region and therefore strains which showed better malolactic activity, are not usually intended to be used as starters, although different authors have proposed the genus *Lactobacillus*, particularly *L. plantarum* species as an alternative to a new generation of malolactic starters [9,28]. Different strains of the species of this genus have shown to be able to develop and carry out the MLF in the hostile conditions of different wines. Among the main drawbacks traditionally attributed to the genus *Lactobacillus*, when considering them as potential starters, include their ability to produce biogenic amines or unpleasant aromas, causing them to be described as spoilage microorganisms. However, later studies showed that these features are dependent on the strain [9,28].

**Figure 2:**
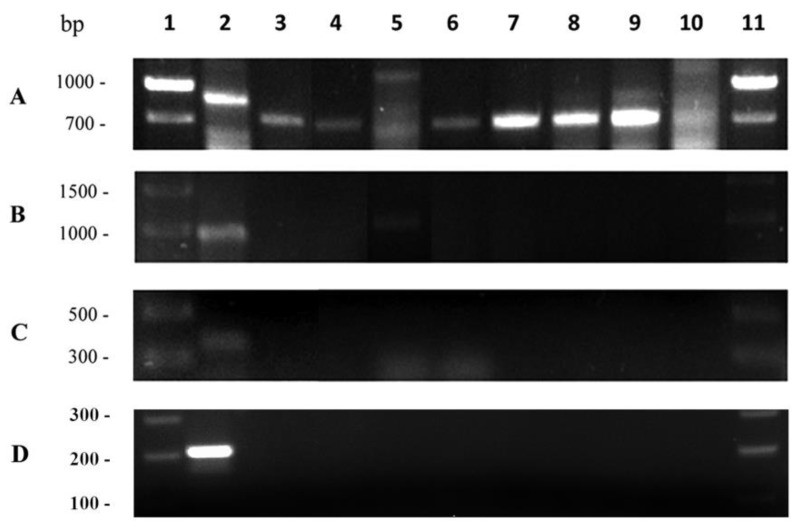
Presence of (**A**) *tdc*, (**B**) *odc,* (**C**) *hdc*, and (**D**) *pdc* genes in LAB strains. Lanes: 1, kb ladder (Bioron); 2, positive control (*Lactobacillus brevis* CECT 5354 for the *tdc* and *pdc* genes, *L. rhamnosus* CECT 288 for the *odc* gene and *L. helveticus* CECT 403 for the *hdc* gene); 3, UVI-14; 4, UVI-20; 5, UVI-5; 6, UVI-17; 7, UVI-18; 8, UVI-21; 9, UVI-23; 10, UVI-2.

Lactic acid bacteria are considered as mainly responsible for the increase of biogenic amines in foods including wines [29]; although LAB strains able to degrade these compounds have also been reported [30]. Biogenic amines, depending on their concentration, can cause numerous adverse health effects [31]. Therefore, this capacity must be tested when proposing them as malolactic starters. The ability to produce the three main biogenic amines present in wines (histamine, tyramine, and putrescine) [31] was tested in the eight strains that showed malolactic activity. Firstly, this capacity was studied at the molecular level by PCR amplification of *hdc* (histidine decarboxylase involved in histamine synthesis route), *odc* (ornithine decarboxylase involved in the synthesis of putrescine), and *tdc* (tyramine synthesis route) genes according to the protocol of De Las Rivas et al. [19]. The results are shown in Figure 2.

An unclear result was found for the *tdc* gene. A band for this gene was amplified in all strains of *L. paracasei* and *L. hilgardii* but with a smaller size than expected as shown by the positive control 2S CECT 5354 (Figure 2A). The *odc* gene was only amplified in the UVI-5 strain and in *L. helveticus* CECT 403 included as a positive control, being absent in the rest of the strains tested (Figure 2B). Finally, the *hdc* gene was not amplified from any of the strains studied comparatively with *L. rhamnosus* strain CECT 288 used as positive control (Figure 2C). These data are consistent with those recently obtained by Nisiotou et al. [32] who also found a very low presence of the gene encoding for histidine decarboxylase in lactic bacteria, including the species *L. hilgardii*.

**Figure 3:**
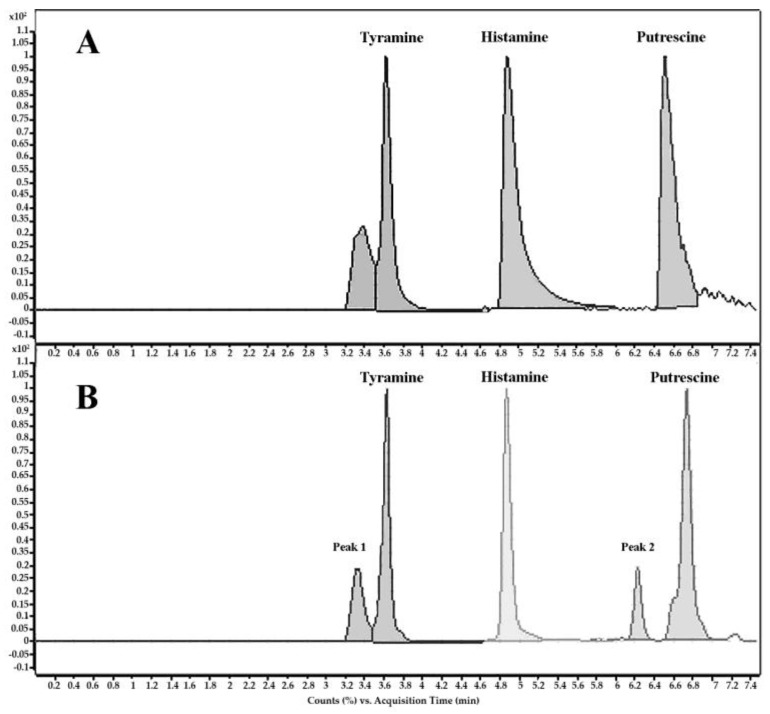
Chromatogram obtained for the analysis of biogenic amines by using UHPLC-MS/MS for: (**A**) mixture of histamine, tyramine, and putrescine standard solution and (**B**) UVI-18 wine sample.

Secondly, to complete and clarify the study, the ability of the above-mentioned eight strains to release biogenic amines in wine was studied by means of LC/MS-MS. The complete separation of the three polar biogenic amines in wine samples was successfully accomplished by using hydrophilic interaction chromatography (HILIC) (Figure 3).

An efficient separation of the three biogenic amines was achieved under the optimized gradient conditions (Rs ≥ 2.8 and α ≥ 1.3). Calibration of the Ultra High Performance Liquid Chromatography coupled with tandem Mass Spectrometer (UHPLC-MS/MS) system was performed under the optimized chromatographic conditions. The results are shown in Table 3. An increase in the levels of tyramine was observed in the case of strains of *L. paracasei* UVI-2 and UVI-5 and in one strain of *L. hilgardii* (UVI-18), but no increase was detected in the remaining strains of *L. hilgardii*. No augmentation on levels of putrescine and histamine was found in any of the strains tested.

For tyramine, only an increase of this amine in wines obtained with three of the strains tested was observed. This result can be explained considering that a significant rise in biogenic amines in wine depends not only on the ability of the strain to produce these compounds but also the concentration of the precursors present in the product [33]. The same explanation would be valid for the case of the strain UVI-5 that shows the *odc* gene and for which no putrescine is detected in wines made from it. This fact also explains that the concentration of biogenic amines is usually higher in red wines than in whites where these precursors usually appear in lower concentrations [34].

The analysis of the results regarding the ability to produce biogenic amines by these studied strains confirms, as previously described [35], that is not a characteristic of the species but varies with the strain, not only to the genus *Lactobacillus*, but also for *O. oeni* strains [28,36], holding the need to study this characteristic for the selection of malolactic starters and confirming the suitability of selected species of the genus *Lactobacillus* to be used as starters.

The influence of MLF on the aromatic composition of wine has been described by different authors [37]. Among the main advantages attributed to the genus *Lactobacillus,* when considering them as potential starters, include its potential ability to synthesize enzymes that can enhance the flavor of wines, differentiating favorably, in this respect, the genus *Lactobacillus* from the genus *Oenococcus*. Two of the enzymes with greater impact on wine aromas are β-glucosidases and phenolic acid descarboxylases. Given that, in the genomes of *L. paracasei* and *L. hilgardii* species, there are genes encoding β-glucosidases, this activity was tested for the eight strains that presented malolactic ability, under unfavorable conditions for the development of MLF in a wine cellar and the results are shown in Table 3. All produced β-glucosidase in varying amounts depending on the strain, highlighting the *L. paracasei* strain UVI-2. Many precursors of aromas are present in must/wine in the form of sensorially imperceptible glycosylated molecules. The activity of β-glucosidases can release the olfactory-active aglycone which contributes to the sensory profile of wines [38]. Consequently, malolactic strains characterized in this study could help to enhance and differentiate the Albariño wine flavor profile in the region studied, providing it with a regional typicality.

Malolactic bacteria can metabolize phenolic acids present in wine [39]. In this case, depending on its concentration, the resulting compound can also add aromatic complexity to the wine or negatively impact its sensory characteristics [22]. Specifically, some studies have questioned the suitability of species of the genus *Lactobacillus* as malolactic starters of interest, because they have the ability to confer unpleasant aromas to the wine due to the production of volatile phenols [22]. De las Rivas et al. [40] found a direct relationship between the presence of the gene encoding a phenolic acid descarboxylase and the ability of the lactic acid bacteria to produce volatile phenols from hydroxycinnamic acids. Moreover, Mtshali et al. [22] showed the presence of the *pad* gene (coding for the enzyme phenolic acid descarboxylase) in the genome of different species of lactic bacteria. The study of the presence of this gene in the genome of malolactic bacteria characterized in this work, compared with *Lactobacillus brevis* strain CECT 5354 used as a positive control (Figure 2D), showed that none of the strains tested carried the *pad* gene. Again, in this case, it was found that it is not a property of the species, but rather the strain, and according to the presence of the *pad* gene, those characterized in this study would not present the risk of adversely affecting the aroma of wine.

## 4. Conclusions

The study of the biodiversity of lactic bacteria associated with musts and wines of the Albariño grape variety, from the region of Val do Salnés in Spain, is presented in this work. The isolates were grouped into four genera and six species: *L. hilgardii*, *L. paracasei*, *L. plantarum*, *L. lactis*, *P. damnosus,* and *O. oeni*. Unlike what has been described for most of the wine regions that describe the *O. oeni* species as a majority, the predominant species in the region studied in this work were *L. hilgardii*, *L. paracasei*, and *L. plantarum*. The ability of sixteen isolates to carry out the MLF under the conditions and characteristics of the wines from this region was evaluated in micro-fermentations. Eight of the strains studied showed a significant capacity to consume malic acid (30–78%) without increasing the levels of acetic acid. These strains were also characterized by their ability to increase the content of biogenic amines in wine showing that five lacked aminogenic capacity and the rest increased in some biogenic amines tested in very small quantities, and, in any case, were much lower than the most restrictive recommended values. Moreover, the ability to produce volatile phenols which may negatively impact the aroma of wine was discarded in the eight strains which showed malolactic activity. Finally, both strains of *L. hilgardii* and *L. paracasei* showed the ability to produce β-glucosidase at different concentrations which could contribute to enhancing and differentiating the aromatic profile of wines by giving them regional typicality. The oenological characterization of the strains, according to the evaluated criteria, showed that the strains of the *Lactobacillus* genus selected in this work (particularly *L. paracasei* UVI-2 and *L. hilgardii* UVI-23) are good candidates to be proposed as new indigenous malolactic starters. Further studies are required to evaluate their suitability for driving malolactic fermentations over time and their behavior at pilot and industrial scale fermentations.

## Figures and Tables

**Table 1:** Identification of lactic acid bacteria (LAB) isolates.

Name	Isolation Year	Sampling Stage *	API^®^ 50 CHL	rDNA 16S Sequencing	16S-ARDRA (Enzyme/s)	ISR Restriction Analysis (Enzyme)	*rec*A Gene Sequencing
*L. plantarum* CECT 220							
*L. paraplantarum* CECT 5787							
UVI-6	2007	MLF	*L. plantarum*/*L. paraplantarum*/*L. pentosus*	*L. plantarum*/*L. paraplantarum*/*L. pentosus*	*L. plantarum*/*L. paraplantarum*/*L. pentosus* (*Bfa*I)	-	*L. plantarum*
UVI-8			*L. plantarum*/*L. paraplantarum*/*L. pentosus*				
UVI-9			*L. plantarum*/*L. paraplantarum*/*L. pentosus*				
UVI-10			*L. plantarum*/*L. paraplantarum*/*L. pentosus*				
UVI-11			-				
*l. casei CECT 475*							
*L. paracasei CECT 4175*							
*L. rhamnosus CECT 288*							
UVI-1	2007	MLF	*L. casei/L. paracasei/L. rhamnosus*	*L. casei/L. paracasei/L. rhamnosus*	*L. casei/L. paracasei/L. rhamnosus* (*Bfa*I, *Btg*ZI)	*L. paracasei* (*Vsp*I)	-
UVI-2			*L. casei/L. paracasei/L. rhamnosus*				
UVI-3			*L. casei/L. paracasei/L. rhamnosus*				
UVI-5			*L. casei/L. paracasei/L. rhamnosus*				
UVI-7			*L. casei/L. paracasei/L. rhamnosus*				
UVI-12			-				
UVI-19			*L. casei/Lb. paracasei/Lb. rhamnosus*				
*L. hilgardii* CECT 4786							
UVI-4	2007	MLF	*L. hilgardii*	*L. hilgardii*	*L. hilgardii* (*Mse*I*, Alu*I*, Hae*III)	-	-
UVI-13							
UVI-14							
UVI-15							
UVI-16							
UVI-17							
UVI-18							
UVI-20							
UVI-21							
UVI-22		AF					
UVI-23		AF					
UVI-26	2008	M					
*O. oeni* CECT 217							
UVI-24	2008	M	-	*O. oeni*	*O. oeni* (*Alu*I*, Bfa*I)	-	-
UVI-25							
UVI-29							
*P. damnosus* CECT 793							
UVI-28	2008	MLF	-	*P. damnosus*	*P. damnosus* (*Alu*I*, Hae*III)	-	-
UVI-31			*P. damnosus*				
UVI-32			-				
UVI-33			*P. damnosus*				
UVI-34			-				
UVI-35			*P. damnosus*				
*L. lactis* CECT 185							
UVI-27	2008	M	*Lc. lactis*	*Lc. lactis*	-	-	-
UVI-30			-				

* CECT: Spanish Type Culture Collection. M: must; AF: alcoholic fermentation; MLF: malolactic fermentation.

**Table 2:** Microvinifications with sixteen LAB strains: malic acid consumption and lactic and acetic acid production.

Strain	Malic Acid	Lactic Acid	Acetic Acid
Control ^a^	3.95 (±0.39)	0.67 (±0.43)	0.21(±0.01)
UVI-2 (*L. paracasei*)	0.98 (±0.11)	3.40 (±0.07)	0.12 (±0.01)
UVI-3 (*L. paracasei*)	3.65 (±0.07)	0.08 (±0.04)	0.22 (±0.00)
UVI-5 (*L. paracasei*)	2.48 (±0.25)	1.95 (±0.28)	0.18 (±0.00)
UVI-9 (*L. plantarum*)	3.48 (±0.04)	0.10 (±0.00)	0.23 (±0.01)
UVI-13 (*L. hilgardii*)	3.58 (±0.04)	0.08 (±0.04)	0.22 (±0.01)
UVI-14 (*L. hilgardii*)	1.23 (±0.04)	3.68 (±0.04)	0.21 (±0.03)
UVI-15 (*L. hilgardii*)	3.55 (±0.07)	0.03 (±0.04)	0.23 (±0.01)
UVI-16 (*L. hilgardii*)	3.55 (±0.07)	0.03 (±0.04)	0.22 (±0.01)
UVI-17 (*L. hilgardii*)	1.03 (±0.04)	4.00 (±0.07)	0.22 (±0.01)
UVI-18 (*L. hilgardii*)	1.05 (±0.00)	3.35 (±0.14)	0.11 (±0.03)
UVI-20 (*L. hilgardii*)	1.18 (±0.04)	3.93 (±0.04)	0.23 (±0.01)
UVI-21 (*L. hilgardii*)	1.10 (±0.14)	3.78 (±0.25)	0.16 (±0.02)
UVI-23 (*L. hilgardii*)	1.08 (±0.11)	3.90 (±0.00)	0.19 (±0.01)
UVI-24 (*O. oeni*)	3.55 (±0.07)	0.03 (±0.04)	0.23 (±0.00)
UVI-25 (*O. oeni*)	3.48 (±0.04)	0.18 (±0.04)	0.23 (±0.01)
UVI-26 (*L. hilgardii*)	4.03 (±0.25)	0.83 (±0.18)	0.33 (±0.01)

Mean values in g/L (±SD); ^a^ uninoculated wine.

**Table 3:** Characterization of selected malolactic strains: β-glucosidase activity, biogenic amine formation capacity, and phenolic acid decarboxylase production.

Strain	Species	β-Glucosidase Activity (U) *	Biogenic Amines Production: Genes (Concentration) ^†^			Phenolic Acid Decarboxylase Gene ^††^
			*hdc*	*odc*	*tdc*	*pad*
UVI-2	*L. paracasei*	36.9 (±1.7)	-	-	+ (0.035)	-
UVI-5	*L. paracasei*	23.6 (±5.0)	-	+	+ (0.065)	-
UVI-14	*L. hilgardii*	14.7 (±0.8)	-	-	+	-
UVI-17	*L. hilgardii*	20.6 (±0.8)	-	-	+	-
UVI-18	*L. hilgardii*	20.6 (±0.8)	-	-	+ (0.152)	-
UVI-20	*L. hilgardii*	23.5 (±5.0)	-	-	+	-
UVI-21	*L. hilgardii*	28.9 (±2.5)	-	-	+	-
UVI-23	*L. hilgardii*	25.3 (±4.2)	-	-	+	-

* Mean values (±SD). ^†^ Presence (+) or absence (−) of the *tdc*, *odc*, and/or *hdc* genes (increase of biogenic amine concentration in mg/L in respect to the control) SD <0.01. ^††^ Presence (+) or absence (−) of the *pad* gene.

## Supplementary Materials

The following are available online at https://www.mdpi.com/2304-8158/9/1/99/s1, Figure S1: Geographical location of the Appellation of Origin Rías Baixas, and position of the subzone of *Val* do Salnés (1), Figure S2: Biochemical profiles of carbohydrate assimilation obtained by using the commercial kit API^®^ 50 CHL, Figure S3: Identification process of isolates of lactic acid bacteria by molecular methods. CECT (Spanish Type Culture Collection), Figure S4: Maximum likelihood phylogenetic tree of (**A**) 1.378 bp 16S rDNA and (**B**) 245 bp *RecA* sequences. Samples, reference strains (CECT), and data base sequences are included. The tree was performed by ClustalW2 and MEGA 5 programs. A bootstrap of 1500 replicates was carried out and values under 70% were omitted, Table S1: Calibration data derived from seven-point calibration for histamine, tyramine, and putrescine using HILIC-MS/MS method for the determination biogenic amines in wine.
