# Supplementary material for: Evaluation of Malolactic Bacteria Associated with Wines from Albariño Variety as Potential Starters: Screening for Quality and Safety
Source: Foods. 2020 Jan 17;9(1):99. doi: 10.3390/foods9010099 (PMC7022644; doi:10.3390/foods9010099)
Supplement: Supplementary file 1 [file foods-09-00099-s001.pdf]

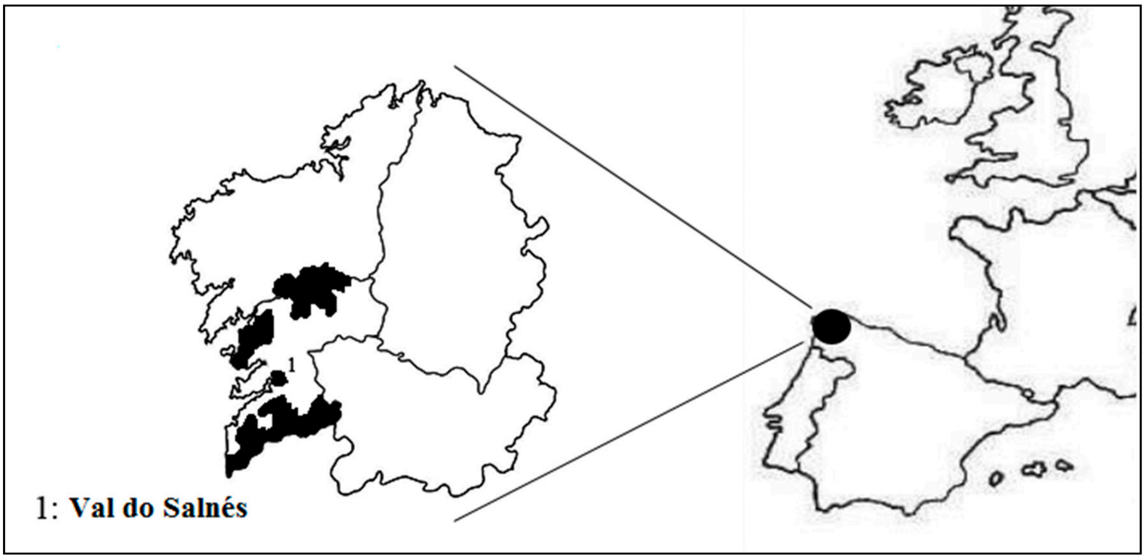

Figure S1: Geographical location of the Appellation of Origin Rías Baixas, and position of the subzone of *Val do Salnés* (1).

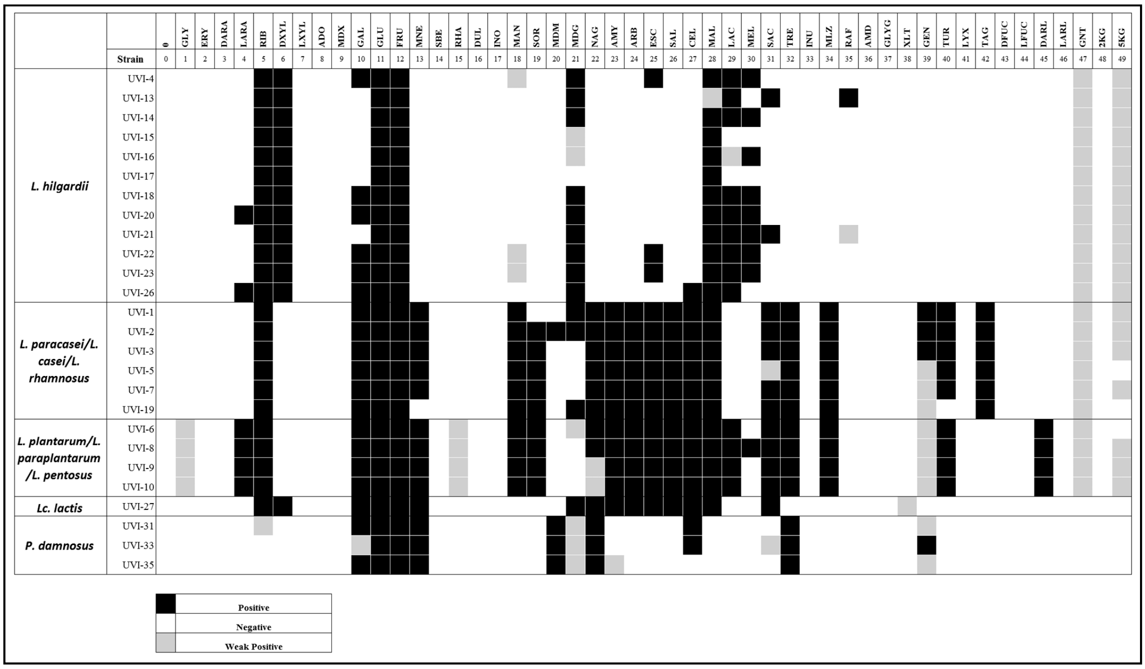

Figure S2: Biochemical profiles of carbohydrate assimilation obtained by using the commercial kit API® 50CHL.

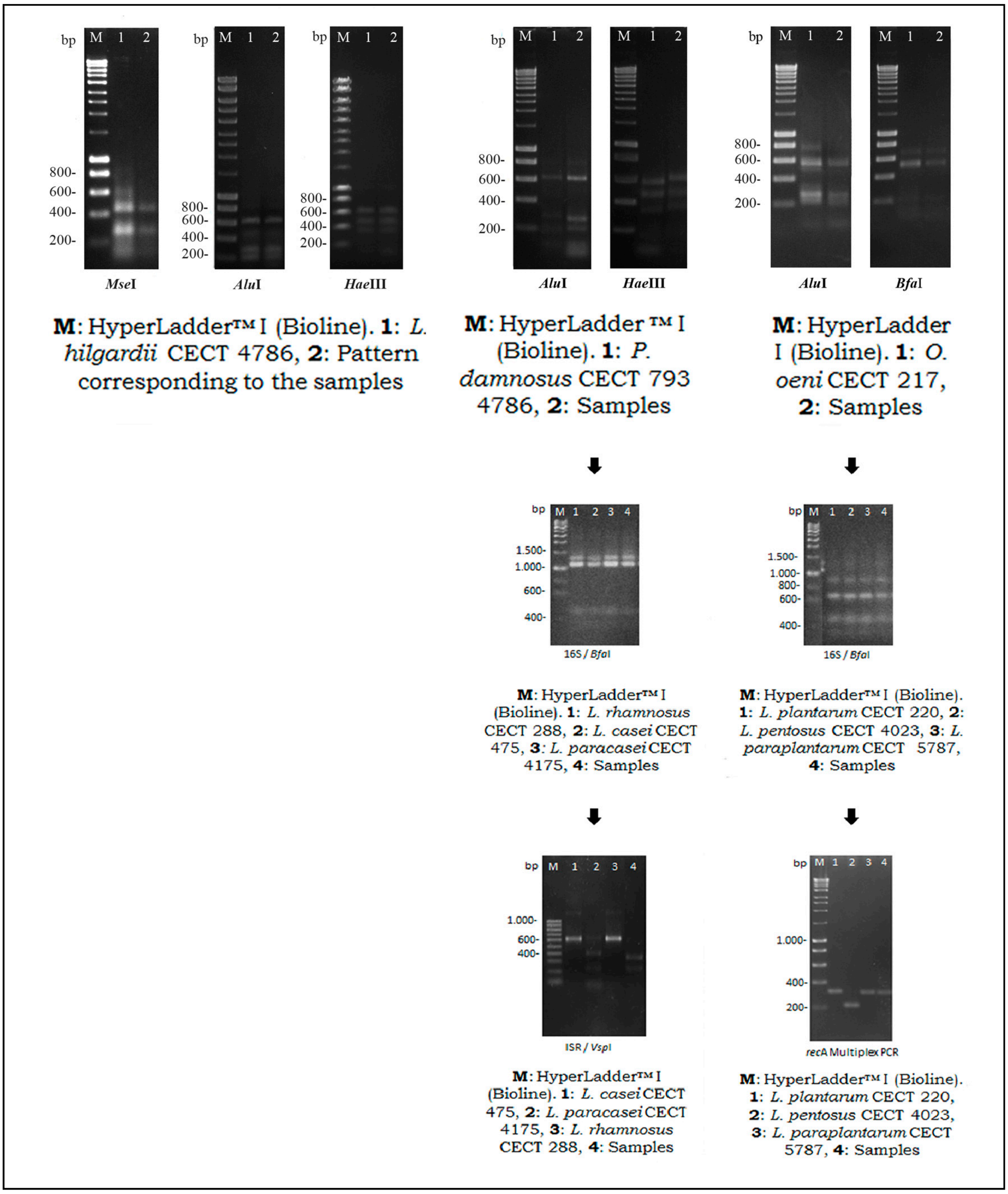

Figure S3: Identification process of isolates of lactic acid bacteria by molecular methods. CECT (Spanish Type Culture Collection).

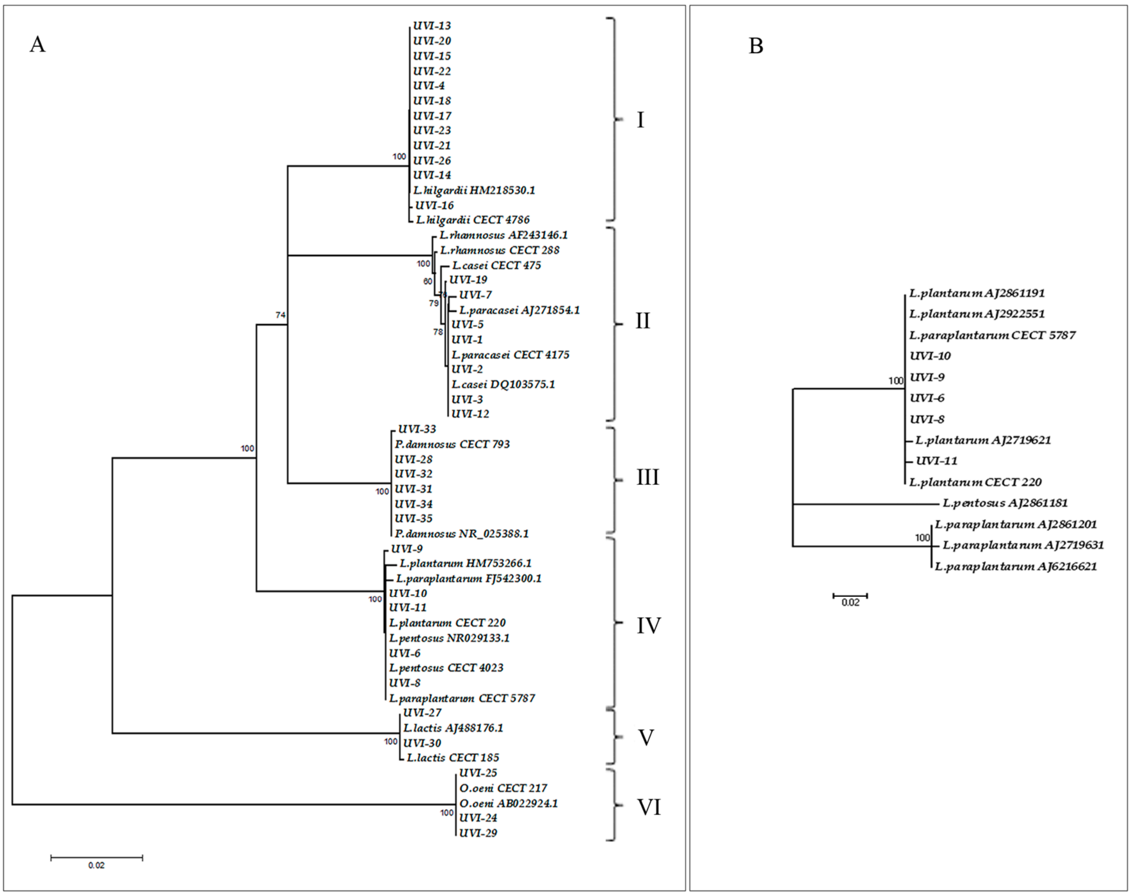

Figure S4: Maximum likelihood phylogenetic tree of 1.378 bp 16S rDNA (A) and 245 bp *RecA* (B) sequences. Samples, reference strains (CECT) and data base sequences are including. The tree was performed by ClustalW2 and MEGA 5 programs. A *bootstrap* of 1.500 replicates was carried out and values under 70% were omitted.

Table S1: Calibration data derived from seven-point calibration for histamine, tyramine and putrescine using HILIC-MS/MS method for the determination biogenic amines in wine.

Compound	Lineal range* (µg/mL)	Slope (m)	Intercept (b)	C. Coefficient (R^2)	LOD (µg/mL)	LOQ (µg/mL)
Histamine	0.25-10	1265111.40	1724726.02	0.9839	0.010	0.034
Tyramine	0.25-10	808162.85	1172034.52	0.9953	0.003	0.010
Putrescine	0.25-10	62363.37	167677.48	0.9939	0.007	0.024

*According to the EU regulatory levels for biogenic amines (10 µg/mL expressed as histamine) the UHPLC-MS/MS method proposed provides adequate LOD and LOQ to carry out the control of biogenic amines in wine samples.

Several wine samples were analyzed using optimal UHPLC-MS/MS conditions before described, three biogenic amines were detected in all samples. Unknown chromatographic peak 1 and 2 also were detected and they shows the same fragmentation patterns as tyramine and putrescine respectively, which mean the presence of some analogues of these two biogenic amines in wine samples used in this study.
